# Application of Deep Learning Model in the Avoidance of Investment Risk of Multinational Enterprises

**DOI:** 10.1155/2022/6578274

**Published:** 2022-06-28

**Authors:** Yan Yan, Guangyu Ye, Fei Feng

**Affiliations:** ^1^School of Business Administration, South China University of Technology, Guangzhou, 510641, China; ^2^Library, Capital Normal University, Beijing, 100048, China

## Abstract

With the continuous improvement and development of the socialist market economic system, China's economic development has full momentum, but the domestic market is no longer sufficient to meet the needs of enterprise development. China has always focused on peaceful diplomacy, and the world market has a strong demand for Chinese products. This work aims to improve the accuracy of exchange rate forecasting. The risk factors that may be encountered in the investment process of multinational enterprises can be effectively avoided. Combining the advantages of Long Short-Term Memory (LSTM) and Convolutional Neural Network (CNN), the LSTM-CNN (Long Short-Term Memory-Convolutional Neural Network) model is proposed to predict the volatility trend of stocks. Firstly, the investment risk of multinational enterprises is analyzed, and, secondly, the principles of the used CNN and LSTM are expounded. Finally, the performance of the proposed model is verified by setting experiments. The experimental results demonstrate that when predicting the 10 selected stocks, the proposed LSTM-CNN model has the highest accuracy in predicting the volatility of stocks, with an average accuracy of 60.1%, while the average accuracy of the rest of the models is all below 60%. It can be found that the stock category does not have a great impact on the prediction accuracy of the model. The average prediction accuracy of the CNN model is 0.578, which is lower than that of the Convolutional Neural Network-Relevance model, and the prediction accuracy of the LSTM model is 0.592, which is better than that of the Long Short-Term Memory-Relevance model. The designed model can be used to predict the stock market to guide investors to make effective investments and reduce investment risks based on relevant cases. The research makes a certain contribution to improving the company's income and stabilizing the national economic development.

## 1. Introduction

Quantitative investment technology has attracted the attention of domestic and foreign investors due to its large market share and stability of income. Especially after entering the 21st century, this technology has developed rapidly in foreign markets. In foreign financial markets, quantitative investment methods have also been widely used [1, 2]. In recent years, professional investors and major financial institutions in China have begun to conduct in-depth research on quantitative investment technology, and quantitative investment funds in the domestic market have also been continuously improved and developed, making domestic investors more diverse when choosing investment products. With the enrichment of investment products, various investment derivatives such as stocks, options, funds, and bonds have emerged in the world. Among them, stock investment has higher risks but can often obtain higher returns. The purpose is to reasonably predict the changing trend of stock prices and avoid investment risks. Traditional econometric and mathematical statistics models predict investment risks of stocks based on stationary sequences. However, since stock market volatility is affected by many factors and the impact mechanism is very complex, it is difficult to meet the forecasting conditions of traditional statistical models [3–5].

Many scholars have studied the stock market through econometrics and mathematical statistics methods such as Autoregressive Moving Average (ARMA) models, multiple regression methods, and exponential smoothing (ES) methods. With the rapid development of artificial intelligence (AI) technologies such as deep learning (DL), it has been widely used in many fields such as machine translation, image processing, and speech recognition and has made breakthroughs. Lv et al. (2021) [6] were also trying to apply DL technology to the financial market to create a scientific quantitative investment system. Model-based investing in financial factors is becoming the dominant approach to quantitative investing. The main challenge is how to choose effective factors to deal with excess market returns. Existing methods, whether selecting factors manually or applying feature selection algorithms, fail to reconcile human knowledge and computing power. Yue et al. (2021) [7] introduced an interactive quantitative investment system that helps stock traders quickly discover promising financial factors from initial recommendations suggested by algorithmic models and jointly refine factors and stocks for the composition of the portfolio. Wang (2019) [8] pointed out a new evolutionary algorithm for automatically generating efficient formulaic Alpha from massive stock datasets. Specifically, the inherent patterns of formulaic letters are first discovered and a hierarchy is proposed to quickly locate promising parts of the search space. Then, a quality diversity search based on principal component analysis (PCA) is proposed to guide the search away from the fully explored space for more desirable results. The current research manifests that the application of DL in the financial field is mainly concentrated in the case of analyzing stock data, and the research on stock trends and countermeasures needs to be improved.

After summarizing the various literature in the world, it can be found that DL technology can be applied to the financial market, and this method can improve the prediction accuracy of stocks. However, most current research works use DL models to simply perform regression classification on stock data and do not consider the time series relationship of stocks. Combining the advantages of Long Short-Term Memory (LSTM) and Convolutional Neural Network (CNN), the LSTM-CNN (Long Short-Term Memory-Convolutional Neural Network) model is proposed to predict the volatility trend of stocks, so that the risks that may be encountered in the investment process can be effectively avoided. The research is mainly divided into four parts. Firstly, it explains the research background and analyzes the research status of the current field. Secondly, the research theory and method are explained, and the neural network (NN) model is designed according to the problem. Thirdly, models are trained and tested to verify the ability of the designed model to handle the problem. Finally, the model is analyzed and summarized through experiments.

## 2. Investment Risk and DL Model Analysis of Multinational Enterprises

### 2.1. Analysis of the Investment Risk of Multinational Enterprises

#### 2.1.1. Country Risk

It refers to the fact that, in the process of foreign investment, due to a series of uncertain factors in the host, such as political corruption and political changes, foreign investors will experience greater economic losses, also known as political risk. Among them, country risk plays an important role. The degree of trade protectionism, industry protection policies, the international relations between the host country and the home country, the relationship between the host country and the surrounding countries, and the differences in relevant laws and regulations constitute the risk factors in the investment process of multinational enterprises [9, 10]. Among them, the factors that have a greater impact on investment activities mainly include the following.

First is the social, political, and economic system risks of the host country, including ideological differences between countries, the integrity of the local government of the investor, policy changes, international religious affairs, and disputes between various parties in the host country. The differences of political groups indicate that there are also differences in the social and economic benefits they represent, so the attitudes and political stances which are taken towards multinational enterprises are also different. Under normal circumstances, the government behavior controlled by democracy in the parliamentary system is relatively standardized, and the laws and decision-making are relatively transparent [11].

Second is the risk of government policy changes and government discriminatory intervention in the host country. To maximize their own interests, the host country often interferes in the behavioral decision-making of corporates. Whether it is a discriminatory intervention or policy intervention, it will bring certain investment risks, which will have a certain impact on the earnings of multinational enterprises.

Third is investment barrier risk and nationalization risk. Among them, the investment barrier risk is mainly related to the market access risk of labor and enterprises. The market access risk generally refers to the host country's restrictions on the entry of products and the scale of foreign investment through certain legal means. Labor risk refers to the risk that foreign personnel enter the country to engage in production operations or investment activities due to the restrictive management of the host country. Due to the corruption of the host country's government, the assets of multinational enterprises may be handed over to the local government of the host country in a plundering manner or at a very low price. The risk caused by this phenomenon is called nationalization risk [12, 13].

#### 2.1.2. Market Risk

The unfavorable impact on enterprises due to the uncertainty of market price fluctuations is called market risk. Fluctuations in commodity market prices and stock markets, changes in benchmark interest rates, and fluctuations in the host country's currency exchange rate will all bring certain risks to the company's operations [14]. As regards interest rate risk, during operation, multinational enterprises will increase their financing costs due to fluctuations in interest rates, and it is difficult to determine the cost and future financing income, which will lead to the loss of their assets. This risk is called interest rate risk. As regards exchange rate risk, in the process of investment, multinational enterprises mainly conduct payment and business transactions in foreign currencies. If the exchange rate of RMB fluctuates during the transaction process, it will bring certain losses to multinational enterprises. This risk is called exchange rate risk. It is mainly related to the exchange rate of the discount price and the transaction price. As regards market price risk, it refers to the fluctuation of product prices under the influence of uncertain factors, which has a certain impact on the supply and demand relationship in the market, and even affects the product sales of enterprises in the local market [15, 16].

Investment risk testifies to the uncertainty of future investment income and may suffer the risk of loss of income or even loss of principal in investment. International risk and market risk are two important sources of enterprise investment risk, which will lead to the occurrence of business losses.

### 2.2. DL Methods

#### 2.2.1. CNN

CNN is mainly composed of the input layer, output layer, fully connected layer, pooling layer, excitation layer, and other functional layers and may also include fusion layer, segmentation layer, and normalization layer [17].

The core layer in the CNN is the convolutional layer, which contains multiple convolution kernels. The local features of the images in the input network can be extracted by different convolution kernels. In general, shallow convolutional layers can only extract low-level local features such as corners and edges, while the extraction of high-level feature images requires deep convolutional layers. The number of convolution kernels, the stride of convolution, the size of the convolution kernel, and the padding are the main parameters of the convolution layer [18–21]. The size of the convolution kernel is set to K  ×  K, the stride of convolution is set to 1, and there is no padding. The obtained convolution calculation is shown in the following equation:

(1) $$\begin{array}{c} c_{i,j}^{s}=\overset{K}{\underset{m=0}{\sum }}\overset{K}{\underset{n=0}{\sum }}W_{m,n}^{s}X_{m+i,n+j}^{s}+b^{S}. \end{array}$$

**Figure 1:**
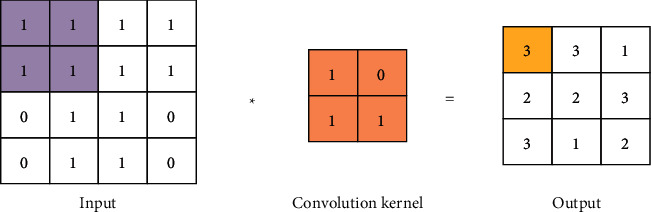
The process of the convolution operation.

In equation (1), *X* represents the single-channel feature map of the input network; *c*_*i*,*j*_ refers to the element of the *i*th row and the *j*th column of the feature map output from the network structure; *W* is the weight value; *b* is the bias value; *S* denotes different biases and weights of different convolution kernels. The convolution operation process is displayed in Figure 1. “Convolution” is actually a mathematical concept that describes the weighted “superposition” of one function and another function in a certain dimension.

**Figure 2:**
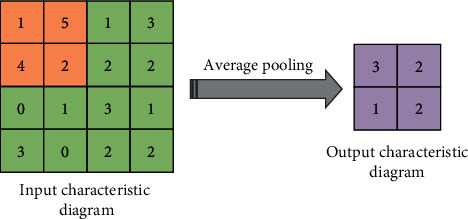
Average pooling.

**Figure 3:**
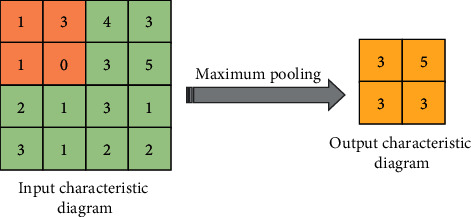
Maximum pooling.

The pooling layer, also known as the downsampled layer, reduces the dimension of the feature map by downsampling, so that the redundant feature information is reduced, and the dimension of the data is reduced. In this process, the quality of the picture will not be affected. In general, the pooling layer can be divided into two types: average pooling and maximum pooling [22, 23]. A schematic diagram of the average pooling operation is expressed in Figure 2. It means dividing the input feature map into multiple regions and calculating the average value as the output. A schematic diagram of the maximum pooling operation is indicated in Figure 3, and it takes the maximum value in each region as the output.

**Figure 4:**
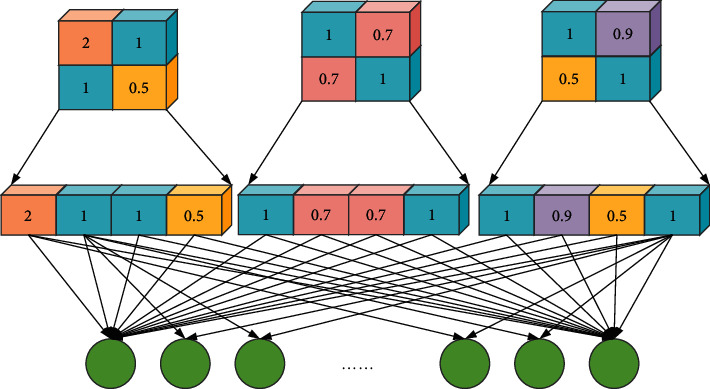
The operation of the fully connected layer.

If the role of the pooling layer and the convolutional layer is to map the original data to the feature space of the hidden layer, then the role of the fully connected layer is to map the learned feature representation to the sample label space, which is equivalent to the “classifier” in the entire CNN [24]. The operation of the fully connected layer is shown in Figure 4. The fully connected layer is the neurons of a single layer, and its neuron nodes are connected to the neuron nodes of the previous layer. It is usually stacked after the convolutional layer in the CNN, and the NN can contain multiple fully connected layers to integrate the extracted feature information.

The activation function plays a very significant role in the composition of the NN. If there is no activation function in the NN, it is no different from a single layer network and can only do some simple linear calculations. The activation function can make the network more flexible to adjust the mapping function, and the function is often a nonlinear function, so the Deep Neural Network (DNN) can fit any complex function.

**Figure 5:**
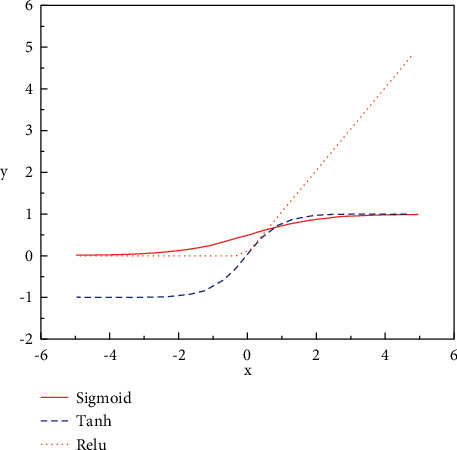
The image of activation functions.

Sigmoid function, Tanh function, and Relu function are widely used activation functions [25–28]. The image of activation functions is exhibited in Figure 5.

The Sigmoid function is the most commonly used activation function in the early classification network, and its expression is shown in the following equation:

(2) $$\begin{array}{c} f\left(x\right)=\frac{1}{1+e^{-x}}. \end{array}$$

The sigmoid function maps the input data between 0 and 1. When the input data is less than 0 and the value is small, the output will be close to 0; when the input data is greater than 0 and the value is large, the output will be close to 1.

The curve structure of the Tanh activation function is similar to that of the Sigmoid function, but the difference between the two is that the Tanh function maps the input data to be between −1 and 1, and the average of the output results is 0, which can speed up the convergence of the network to a certain extent. When the input value is small or large, the problem of vanishing gradient still occurs, resulting in stagnation of training. The expression of the Tanh activation function is shown in the following equation:

(3) $$\begin{array}{c} y=\frac{e^{x}-e^{-x}}{e^{x}+e^{-x}}. \end{array}$$

The Sigmoid activation function has been gradually replaced by the Relu function in recent years and has been widely used in various networks. The Relu activation function has two main advantages. One is to effectively solve the problem of vanishing gradient of the network caused by the Tanh function and the Sigmoid function. When the input data is positive, the gradient can always be 1. Another is that the convergence efficiency of the Relu activation function is significantly improved compared with the Tanh function and the Sigmoid function [29]. The expression of this function is shown in the following equation:

(4) $$\begin{array}{c} y=\left\{\begin{array}{c} x,x\geq 0,\\ 0,x<0. \end{array}\right. \end{array}$$

It can also be expressed by the following equation:

(5) $$\begin{array}{c} y=\text{max}\left(0,x\right). \end{array}$$

#### 2.2.2. LSTM

**Figure 6:**
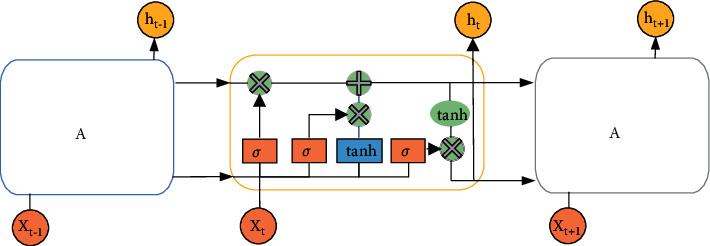
The structure of memory cell of LSTM.

As early as 1997, Hochreite et al. proposed a LSTM model. LSTM is actually an improvement of the Recurrent Neural Network (RNN) model. RNN is a general term for a series of NNs that can process time series data and can process sequence data of any length. However, due to the vanishing or explosion gradient of RNN, it can only learn short-period dependencies, and, in practice, to reduce complexity, it is often assumed that the current state is only related to several previous states [30, 31]. To solve this problem, the researchers proposed the LSTM structure, which contains a set of memory units to record all the historical information up to the current moment and is controlled by the input gate, the forgetting gate, and the output gate. The input gate is mainly to control the amount of new information added to each memory unit, the forget gate is mainly to control the amount of forgotten information of each memory unit, and the output gate is mainly to control the amount of output information of each memory unit. The structure of memory cell is shown in Figure 6.

In Figure 6, the output vector of the previous timestamp and the input of the current timestamp pass through the activation function Tanh to obtain a new output vector. *σ* is the Sigmoid activation function. On the basis of this chain, LSTM improves the interior of the module and uses three Sigmoid NN layers and a gate composed of point-by-point multiplication operations to strengthen the ability to control information. The Tanh activation function mainly processes the data for the state and output functions. The forget gate determines which information needs to be discarded in the cell state. It is the first step of the memory cell of the LSTM, and its element value is between (0, 1). The specific expression is shown in the following equation:

(6) $$\begin{array}{c} f_{t}=\sigma \left(W_{f}\cdot \left[h_{t-1},x_{t}\right]+b_{f}\right). \end{array}$$

In equation (6), *h*_*t*−1_ indicates the output value of the previous cell; *x*_*t*_ means the input value of the current cell.

The second step of the memory cell of the LSTM is to determine how much new information needs to be added to the cell state. The specific expression is shown in the two following equations:

(7) $$\begin{array}{c} i_{t}=\sigma \left(W_{i}\cdot \left[h_{t-1},x_{t}\right]+b_{i}\right), \end{array}$$

(8) $$\begin{array}{c} {\tilde{C}}_{t}=\text{tan}\;\;h\left(W_{C}\cdot \left[h_{t-1},x_{t}\right]+b_{C}\right). \end{array}$$

In equations (7) and (8), *W*, *b* represent the weight and offset of the threshold layer, respectively. After the update of each threshold layer is completed, the memory cell *C*_*t*_ is updated again. The calculation is shown in the following equation:

(9) $$\begin{array}{c} C_{t}=f_{t}\times C_{t-1}+i_{t}\times C_{t}. \end{array}$$

The main function of the output gate is to calculate the output value of the cell. The output value of the previous moment, the input value of the cell at the current moment, and the cell state together determine the size of the cell output value. The specific expression is shown in the two following equations:

(10) $$\begin{array}{c} o_{t}=\sigma \left(W_{o}\cdot \left[h_{t-1},x_{t}\right]+b_{o}\right), \end{array}$$

(11) $$\begin{array}{c} h_{t}=o_{t}\times \;\;\tan\;\;h\left(C_{t}\right). \end{array}$$

The structure of LSTM is actually based on the RNN, adding the filtering of the past state, and learning the weights and offsets of each threshold layer from the past information. In the real-time prediction stage, the trained model is used to operate the input data to obtain the predicted value of the time series, thereby improving the efficiency of mining past information and shortening the training time [32, 33].

### 2.3. The Setting of the Experiment

#### 2.3.1. Experimental Datasets and Test Indicators

In this experiment, the constituent stocks with strong liquidity and large market size in Shanghai Securities are taken as the research objects to analyze the market correlation, and the experimental data used are from Shanghai Securities. The research period of this experiment is from February 1, 2015, to April 27, 2020, and the period from February 1, 2015, to April 30, 2019, is divided into the training set of this experiment, and the period from May 1, 2019, to April 27, 2020, is divided into the test set. Two feature sets of quantitative and price feature and related income features are constructed. Due to the existence of missing values in the data and high noise, the prediction accuracy and training speed of the model will be affected. Therefore, the original data needs to be preprocessed first.

Missing values are handled by removing data entries with missing values. Meanwhile, to eliminate the influence of each index, it is necessary to normalize the data. This experiment uses the Z-Score normalization method to normalize the data input features. The Z-Score normalization method is also called the standard deviation normalization method. The standard deviation of the data processed by this method is 1, the mean is 0, and the transformation function is written in the following equation:

(12) $$\begin{array}{c} \bar{x}=\frac{x-\mu }{\sigma }. \end{array}$$

The market generally believes that the amount of trading volume will affect the rise and fall of stock prices. The quantitative and price feature is to predict the trend of stock fluctuations based on information such as trading volume, opening price, and closing price. The experiment predicts the fluctuation trend of the stock price of the next day through the stock data of the first 30 days, so that the quantitative and price feature of the stock can be obtained, as demonstrated in the two following equations:

(13) $$\begin{array}{c} {\text{feature}}_{t}^{s}=\left[{\text{price}}_{t-29,}^{s}{\text{price}}_{t-28}^{s},\ldots ,{\text{price}}_{t}^{s}\right], \end{array}$$

(14) $$\begin{array}{c} {\text{prices}}_{t}^{s}=\left[{\text{open}}_{t},{\text{ high}}_{t},{\text{ close}}_{t},{\text{low}}_{t},{\text{volumn}}_{t},{\text{amount}}_{\text{t}}\right]. \end{array}$$

Here, prices_*t*_^*s*^ means the quantitative and price feature of stock *s* on day *t*; open_*t*_ indicates the opening price on day *t*; high_*t*_ demonstrates the highest value on day *t* price; close_*t*_ manifests the closing price on day *t*; low_*t*_ represents the lowest price on day *t*; volumn_*t*_ is the trading volume on day *t*; amount_*t*_ refers to the transaction amount on day *t*.

The historical price of a single stock not only affects its own future price but also affects the future price of other stocks related to it. Therefore, S stocks with strong correlation are used to build their relevant income characteristics, as shown in the two following equations:

(15) $$\begin{array}{c} {\text{feature}}_{t}=\left[{\text{return}}_{t-29},{\text{ return}}_{t-28},\ldots ,{\text{return}}_{t}\right], \end{array}$$

(16) $$\begin{array}{c} {\text{return}}_{t}=\left[{\tilde{r}}_{t}^{1},{\tilde{r}}_{t}^{2},\ldots ,{\tilde{r}}_{t}^{S}\right]. \end{array}$$

return_*t*_ refers to the return list of S stocks on day *t*.

#### 2.3.2. Model Design and Parameter Optimization

**Figure 7:**
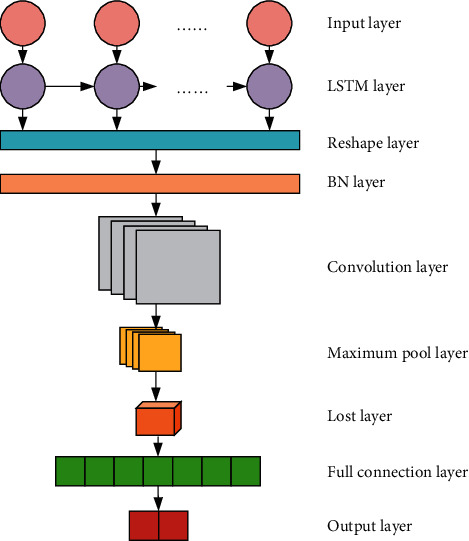
LSTM-CNN structure.

**Figure 8:**
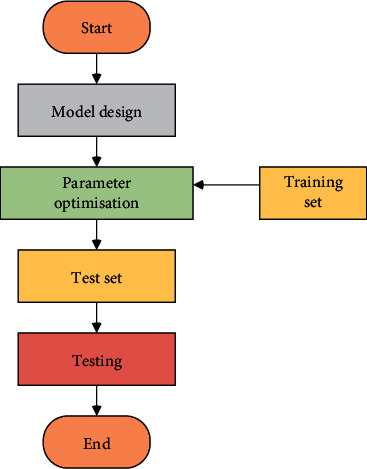
Flow chart of the experiment.

Considering the advantages of the CNN model and the LSTM model, the LSTM-CNN structure is established, and the Batch Normalization (BN) layer is used as a transition layer to adapt to the problem of stock risk prediction. Its network structure is displayed in Figure 7. The training process of the model is expressed in Figure 8, and the specific settings of the network structure are illustrated in Table 1.

## 3. Analysis of the Results of the Experiment

### 3.1. Comparison of Market Structures Based on Stock Correlations

**Figure 9:**
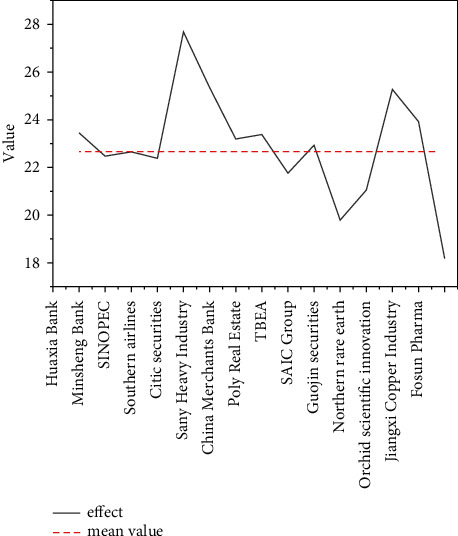
The statistical results of influence.

The market correlation analysis is based on the logarithmic difference return of the stock closing price from January 1, 2015, to April 1, 2020. During the experiment, 0 is used to fill in the missing values to simplify the calculation process. The sum of correlations is represented by influence, and the group with a larger influence value indicates that the stock has a greater influence on other stocks. The statistical results of the influence of the 15 stocks participating in this experiment are illustrated in Figure 9. The impact indicators in the figure are commonly used research indicators.

In Figure 9, among the 15 stocks participating in this experiment, 8 stocks have a higher influence than the average, and it shows that, except for the two stocks of Northern Rare Earth and Fosun Pharma, which have extremely low influence on the market, most of the other stocks will have a certain impact on the stock market. If the influence of the stock is higher than the average, it is considered that the stock will have a greater impact on the market; otherwise, it is defined as a stock with less influence on the market.

### 3.2. Comparison of Stock Trend Forecasting Models

To verify the effectiveness of the proposed LSTM-CNN model, this experiment uses 5 financial stocks and industrial stocks with strong market influence as datasets for verification. The prediction effects of six models, LSTM-CNN, LSTM, CNN, Random Forest (RF), Convolutional Neural Network-Relevance (CNN-R), and Long Short-Term Memory-Relevance (LSTM-R), are analyzed. The selected stocks are listed in Table 2.

**Figure 10:**
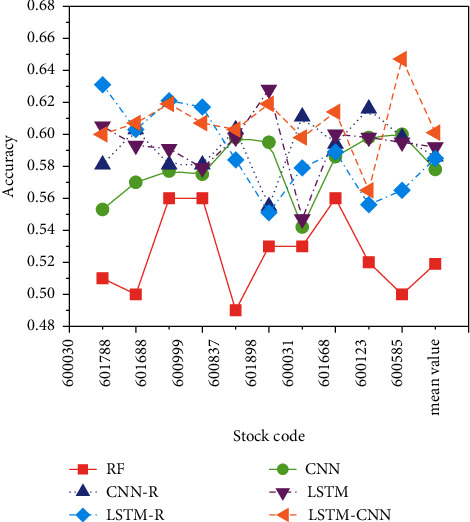
The results of the prediction accuracy.

The accuracy refers to the proportion of correctly classified samples in the total samples. In the case of unbalanced data distribution, a high accuracy does not reflect the superiority of an algorithm, but the accuracy indicator is indeed very intuitive. The effect of model predictions can be evaluated as a whole. The results of the prediction accuracy of each model for the selected stocks are expressed in Figure 10.

In Figure 10, the proposed LSTM-CNN model has the highest accuracy in predicting the volatility of stocks, with an average accuracy of 60.1%, while the average accuracy of the rest of the models is all below 60%. It indicates that the stock category does not have a great impact on the prediction accuracy of the model. The average prediction accuracy of the CNN model is 0.578, which is lower than that of the CNN-R model, and the prediction accuracy of the LSTM model is 0.592, which is better than the LSTM-R model. The nature of the model itself causes this difference. The characteristic of the CNN model is that the input features are extracted through the convolutional layer, and the correlation input provides more information for the CNN model, which makes the role of the convolution layer significantly improved.

**Figure 11:**
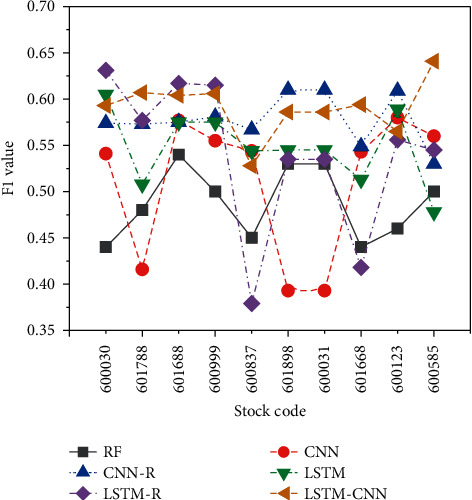
The model predicts the F1 value of the up and down categories.

An important indicator for measuring classification problems is the F1 value, which is the harmonic mean of recall and precision, and it is often used for final evaluation of multiclassification problems. Forecasting both up and down stocks is vital in the stock forecasting problem, and predicting down stocks as up stocks will not only reduce profits but also may even cause property loss. The F1 values predicted by each model structure for the up and down categories are indicated in Figure 11.

In Figure 11, when predicting the 10 selected stocks, the constructed LSTM-CNN model has the best prediction effect, with an average F1 value of 0.585, followed by the LSTM model with an average F1 value of 0.567. Overall, the RF model performs poorly on the classification ability of prediction, and it is indicated that the stock category does not have a large impact on the prediction performance of the model. The characteristic of the LSTM model is that it can record historical information and learn the relationship between time series, and the characteristic of the correlation is the return of multiple stocks. For the LSTM model, it contains too much irrelevant information, and it is difficult to identify the information that is more important to the prediction result.

## 4. Conclusion

With the development of economic globalization, this not only brings opportunities for countries in the world to invest overseas but also makes them face risks and challenges in the process of overseas investment. Compared with western developed countries, China's foreign development started relatively late and lacks experience. In the process of investment and development, it is inevitable to encounter various difficulties and risks. Combining the advantages of LSTM and CNN, the LSTM-CNN model is proposed to predict the volatility trend of stocks. Firstly, the investment risk of the multinational enterprises is analyzed, and, secondly, the principles of the used CNN and LSTM are expounded. Finally, the performance of the proposed model is verified by setting experiments. The experimental results reveal that the proposed LSTM-CNN model has the highest accuracy in predicting the volatility of stocks, with an average accuracy of 60.1%, while the average accuracy of the rest of the models is less than 60%. The constructed LSTM-CNN model has the best prediction performance, with an average F1 value of 0.585.

The research content will help to promote China's large multinational enterprises to avoid risks, enhance benefits, and enhance their international competitiveness. It will play a significant supporting role in realizing the “going global “strategy, assisting the common development of the “Belt and Road,” and implementing the national foreign economic strategy of mutually beneficial cooperation and win-win. This will have a vital supporting role and guiding significance for promoting domestic enterprises to going global and promoting the transformation and upgrading of market structure. The constructed LSTM-CNN only includes a single LSTM layer and a convolutional layer. Although the proposed network model has strong advantages in prediction accuracy and profitability, in the follow-up work, the use of multiple LSTM layers or convolutional layers can still be considered to further optimize the network structure.

## Figures and Tables

**Table 1:** The settings of the network structure.

Floor number	Name	Output dimension	Other settings
0	Input layer	(batch, 30, 6)	Time step: 30
1	LSTM layer	(batch, 30, 32)	Recurrent dropout = 0.25 Dropout = 0.2
2	Reshape layer	(batch, 30, 32, 1)	
3	BN layer	(batch, 30, 32, 1)	
4	Convolution layer	(batch, 30, 32, 32)	Convolution kernel size = 3 *∗* 3
5	Maximum pool layer	(batch, 15, 16, 32)	
6	Lost layer	(batch, 7680)	
7	Full connection layer	(batch, 2)	
8	Output layer	(batch, 30, 32, 1)	

**Table 2:** The selected stocks.

Stock code	Stock name
600030	CITIC Securities
601788	Everbright Securities
601688	Huatai Securities
600999	China Merchants Securities
600837	Haitong Securities
601898	China Coal Energy
600031	Sany Heavy Industry
601668	Chinese architecture
600123	Orchid Science and Technology
600585	Conch Cement

## Data Availability

The data used to support the findings of this study are included within the article.
